# Posterior mitral annuloplasty for enhancing mitral leaflet coaptation: using a strip designed for placement in the posterior annulus

**DOI:** 10.1186/s13019-015-0350-6

**Published:** 2015-11-13

**Authors:** Jong Hun Kim, Kyung Hwa Kim, Jong Bum Choi, Ja Hong Kuh

**Affiliations:** 1 Department of Thoracic and Cardiovascular Surgery, Chonbuk National University Medical School, 20 Geonji-Ro, Deokjin-Gu, Jeonju, Chonbuk 561-712 Republic of Korea; 2 Research Institute of Clinical Medicine of Chonbuk National University and Biomedical Research Institute of Chonbuk National University Hospital, Jeonju, Chonbuk 561-712 Republic of Korea

**Keywords:** Heart valve, Mitral valve repair, Surgery/techniques

## Abstract

**Background:**

In patients with mitral valve regurgitation (MR), posterior mitral annuloplasty (PMA) was performed for mitral valve repair using a strip designed for placement in the posterior annulus, sparing the anterior annulus and anterior half of the commissures.

**Methods:**

Between September 2009 and October 2013, we performed PMA using a novel strip in 74 consecutive patients with MR greater than 3+. Procedures associated with mitral valve repairs were performed in 41 patients (56.9 %), including new chord placement for leaflet prolapse (*n*=30), patch valvuloplasty for posterior chord rupture (*n*=4), and posterior leaflet augmentation (*n*=15). All patients were analyzed by serial echocardiographic follow-up, and preoperative and postoperative computed tomography was performed in 10 randomly selected patients.

**Results:**

Hospital death occurred in two patients (2.7 %), and 72 survived patients were completely followed up. At a mean follow-up of 37.2 ± 15.0 months, the MR grade was zero or 1+ in 64 patients (88.9 %), 2+ in 7 patients (9.7 %), and 3+ in one patient (1.4 %). The mean indexed valve area and mean valve gradient were 1.7 ± 0.4 cm^2^/m^2^ and 3.5 ± 1.2 mmHg, respectively. The mean leaflet coaptation height in early systole was 12.8 ± 3.5 mm. During the cardiac cycle, the repaired valves exhibited dynamic changes of 19.5 ± 9.3 % in the septo-lateral dimensions. No early conversions to valve replacements or late reoperations occurred. None of the patients with remnant or recurrent MR experienced hemolysis.

**Conclusions:**

PMA using a novel strip showed a sufficient coaptation height secondary to reduction of the septo-lateral annular dimensions and dynamic changes in the dimensions. It can be expected to be an alternative mitral annuloplasty technique with satisfactory results.

## Background

Most remodeling annuloplasty rings fix the annular dimensions of the mitral valve (MV) in a settled shape [1]. The partial flexible bands restrict the hinge motion between the anterior and posterior leaflets due to their fixation to trigones, commissures, and the posterior annulus. The rings and bands can restore valve competence, but they restrict most annular motion. In addition, because the annulus is fixed to the ring or band in a flat plane, it loses the commissural hinge work and three-dimensional saddle shape [2, 3]. Such normal annular geometry may be preserved by an annuloplasty strip that spares the anterior annulus and commissures. We retrospectively evaluated in a cohort of patients underwent posterior mitral annuloplasty (PMA) using a novel strip designed for placement in the posterior annulus.

## Methods

This study was a retrospective review of the prospective follow-up of mitral valve regurgitation (MR) patients who underwent PMA using a novel annuloplasty strip for MV repair. This study was approved by the Institutional Review Board at Chonbuk National University Hospital.

### Patients

From September 2009 to October 2013, a total of 74 consecutive patients (38 men and 36 women; mean age, 61.5±14.2 years) with MR greater than 3+ underwent PMA using a strip (Table 1). Procedures associated with MV repair were performed in 41 patients (56.9 %): new chord placement with anterior leaflet prolapse in 22 patients (29.7 %), posterior leaflet prolapse in 15 patients (20.3 %), and commissural prolapse in 3 patients (4.1 %), patch valvuloplasty [4] with posterior chord rupture in 4 patients (5.4 %), and posterior leaflet augmentation with tethered or short posterior leaflet in 15 patients (20.3 %). Concomitant procedures included the Cox-Maze procedure (*n*= 28), aortic valve replacement or repair (*n*=21), coronary artery bypass grafting (*n*=9), and ascending aortic aneurysm repair (*n*=11, resection in 2 and wrapping in 9).

**Table 1:** Preoperative characteristics of 74 patients undergoing PMA for mitral valve regurgitation

Characteristic	Value
Male sex	38 (51.4)
Age, years	61.5±14.2
BSA, M^2^	1.62 ± 0.19
NYHA III and IV	51 (68.9)
Preop LVEF, %	54.3 ± 10.5
PAP, mmHg	41.8 ± 15.7
Cause of MR	
Degenerative	55 (74.3)
Rheumatic	7 (9.5)
Ischemic	7 (9.5)
Acute chord rupture	4 (5.4)
Chronic inflammation (myeloproliferative)	1 (1.4)
Preop MR grade	
3+ (moderate to severe)	34 (45.9)
4+ (severe)	40 (54.1)

Data are n (%) or mean ± SD

*BSA* body surface area, *LVEF* left ventricular ejection fraction, *MR* mitral valve regurgitation, *PAP* pulmonary artery pressure, *Preop* preoperative

### Posterior mitral annuloplasty strip

**Fig. 1:**
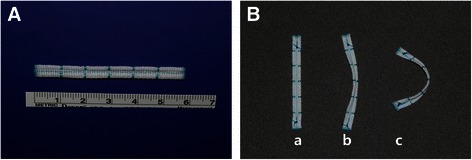
**a** A posterior mitral annuloplasty strip with two thick margins and a thin middle valley. **b** Although the flexible strip is flat when it is straight (**a**), it is lifted in the middle portion by its characteristic structure when placed in a curve in the posterior annulus (**b** and **c**)

The PMA strip (Mitra-Lift® strip, Scien-City, Inc., Seoul, ROK) (Fig. 1A) is a flat Dacron strip of 5.0 mm in width with two thick margins and one thin middle gully. The strip is flat when straight, but the middle portion is lifted when it is curvilinear (Fig. 1B). The strip is placed along the posterior annulus, sparing the anterior annulus and anterior half of both commissural annuli. Placement of the strip on the atrial wall plane along the posterior annulus results in a curvilinear complex that lifts the middle portion of the posterior annulus (Fig. 1B-b). The middle portion of the posterior annulus becomes the highest posterior horn of the posterior mitral annulus. Because the commissural angles are not fixed, the commissural hinges between two leaflets can be expected to be preserved. In addition, because the strip tends to be straightened by its two thick margins, the septo-lateral annular dimensions may be reduced and the commissural hinges made. The strip length was determined by multiplying by about 1.5 times the anterior annular length. The strip lengths used were 53, 55, 58, and 61 mm.

### Surgical procedure

**Fig. 2:**
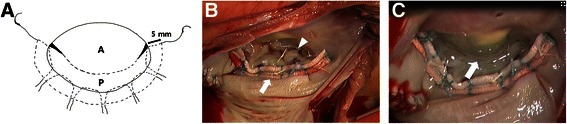
**a** The strip is placed using six interrupted 2–0 Dacron mattress sutures that are passed through the 5-mm left atrial wall and posterior annulus. **b** For mitral regurgitation due to commissural chordal rupture, new chord placement (*white arrowhead*) and strip annuloplasty (*white arrow*) were performed. **c** In the saline test, the leaflet coaptation (*white arrow*) was placed below the strip. A: anterior leaflet; P: posterior leaflet

The PMA procedure has been described in detail previously [5]. The MV was exposed under cardioplegic arrest with moderate hypothermic cardiopulmonary bypass. Prior to PMA, new chord placement was performed for anterior or posterior leaflet prolapse [6]. After a suitable strip size was determined, six braided 2–0 Dacron sutures were passed in inverted U-shapes through the supra-annular atrial wall (5 mm in length) and the posterior annulus from commissure to commissure (Fig. 2a). Both end sutures were placed at the middle portion of the commissural annulus. All sutures were passed through the middle gully of the strip and tied. After the strip was placed on the atrial wall along the posterior annulus, the annulus and strip became curvilinear due to the circular force of the annulus (Fig. 2b). In a saline test, the leaflet coaptation line was located below the strip, not at strip level (Fig. 2c). In cases of tethered posterior leaflets associated with rheumatic valve disease or ischemic MR or a narrow posterior leaflet < 10 mm in height, a sufficient leaflet area for coaptation was created by posterior leaflet augmentation. The posterior leaflet was detached from the posterior annulus, leaving 3-5 mm of intact leaflet tissue at both ends of the posterior leaflet, and the defect was augmented with an elliptical bovine pericardial patch 15 mm × 45 mm using running 5–0 polypropylene sutures.

### Computed tomography

In 10 randomly selected patients, preoperative and postoperative computed tomography was performed to observe the mitral annular shape.

### Echocardiographic measurements

Transthoracic echocardiography was performed at admission, discharge, 6 months postoperatively, and annually. The MR grade was determined according to the following scale: 0, no or trivial MR; 1+, mild; 2+, mild to moderate MR; 3+, moderate to severe MR; and 4+, severe MR. The MV orifice area was assessed by the pressure half-time method. In the parasternal long-axis view, the maximum and minimum septo-lateral dimensions were measured in diastole and systole, respectively. The coaptation height (i.e., the longest coaptation length of the anterior and posterior leaflets) was measured in early systole.

### Statistical analysis

All statistical analyses were performed in SPSS 18.0 (IBM, Armonk, NY, USA). Continuous variables were expressed as the mean ± standard deviation and compared using the Student’s t test and paired t-tests. Categorical variables were expressed as proportions (%) and compared using the χ^2^ test.

## Results

**Fig. 3:**
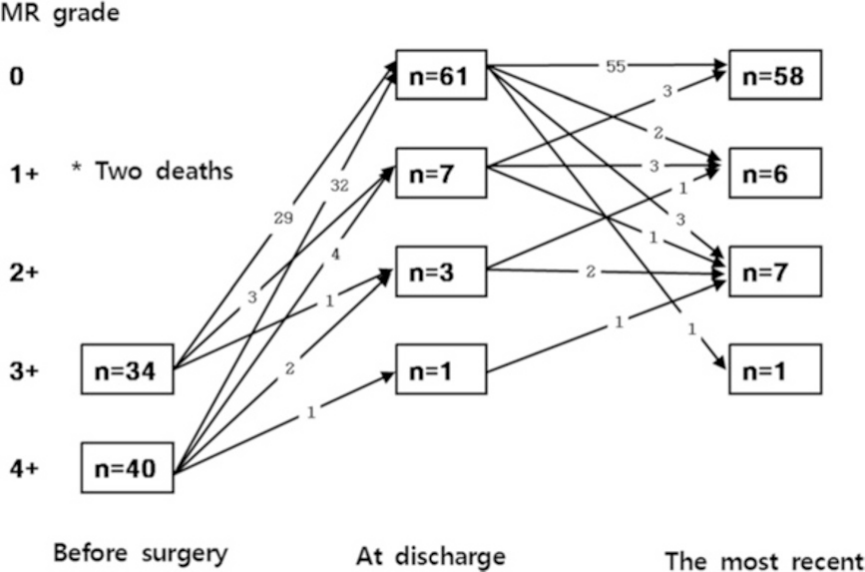
Serial changes in mitral regurgitation after posterior mitral annuloplasty

**Fig. 4:**
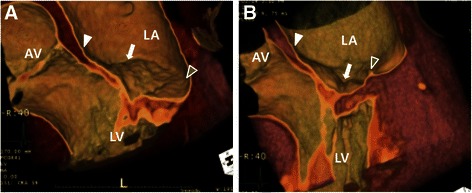
Preoperative (**a**) and postoperative (**b**) computed tomographic study of a patient who underwent posterior mitral annuloplasty for mitral regurgitation secondary to annular dilation. **a** A preoperative sagittal view showed that the medial commissural annulus (white arrow) was placed in the plane between the anterior annulus (*white arrowhead*) and posterior annulus (white empty arrowhead). **b** The postoperative sagittal view showed that the hinged medial commissure (*white arrow*) was placed below the antero-posterior annular plane. AV: aortic valve; LA: left atrium; LV: left ventricle.

Hospital death occurred in two patients (2.7 %) due to pneumonia on postoperative day 32 and 93. The mean follow-up was 37.2 ± 15.0 months. All survived patients were in NYHA class I or II. In follow-up echocardiographic study of 72 patients, the most recent MR grade was zero in 58 patients (80.6 %), 1+ in 6 patients (8.3 %), 2+ in 7 patients (9.7 %), and 3+ in one patient (1.4 %) (Table 2). No significant progression of the MR grade was noted during the follow-up period (comparison of MR grades at discharge and the latest follow-up; *p* = 0.240; Fig. 3). In computed tomographic study, preoperatively the commissural hinge was placed at the flat annular plane (Fig. 4a). Postoperatively, however, the hinge was located below the antero-posterior annular plane (Fig. 4b).

**Table 2:** Postoperative data

Characteristic	Value
MR grade, at discharge	
0 (absent or trivial)	61 (81.4)
+1 (mild)	7 (9.7)
+2 (moderate)	3 (4.2)
+3 (moderate to severe)	1 (1.4)
+4 (severe)	0 (0)
MR grade, the latest	
0 (absent or trivial)	58 (80.6)
+1 (mild)	6 (8.3)
+2 (moderate)	7 (9.7)
+3 (moderate to severe)	1 (1.4)
+4 (severe)	0 (0)
MVA, cm^2^	2.7 ± 0.5
Index MVA, cm^2^/m^2^	1.7 ± 0.4
Valve gradient, mmHg	3.5 ± 1.2
Leaflet coaptation height, mm	12.8 ± 3.5

Data are *n* (%) or mean ± SD

*MVA* mitral valve orifice area, *MR* mitral valve regurgitation

**Fig. 5:**
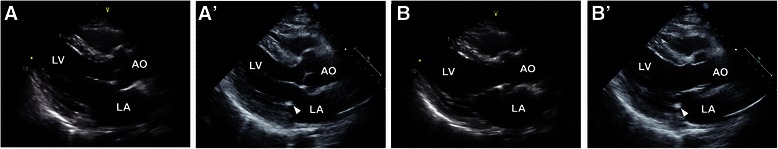
Preoperative (**a** and **b**) and postoperative (**a’** and **b’**) echocardiograms of a patient who underwent new chordae placement for posterior leaflet prolapse and posterior mitral annuloplasty. Parasternal long-axis views showed that the septo-lateral dimensions were reduced after posterior mitral annuloplasty (**a’** and **b’**, arrowhead) in diastole (**a**-**a’**) and systole (**b**-**b’**). AO: aorta; LA: left atrium; LV: left ventricle

On echocardiography, the septo-lateral dimensions were decreased after PMA (Fig. 5a and a’, b and b’) and the coaptation was located below the strip (Fig. 5b’). In the echocardiographic follow-up, the MV area and MV area index were 2.7 ± 0.5 cm^2^ and 1.7 ± 0.4 cm^2^/m^2^, respectively, and the mean transvalvular pressure gradient was 3.5 ± 1.2 mmHg. The mean leaflet coaptation height in early systole was 12.8 ± 3.5 mm (Table 2). During the cardiac cycles, the ratio of the maximum and minimum septo-lateral dimensions postoperatively was 19.5 ± 9.3% (minimum, 17.6 ± 4.3 mm vs. maximum, 21.1 ± 5.1 mm; *p* < 0.0001; Table 3). None of the patients with mild to moderate MR experienced hemolysis. No patients required edge-to-edge repair, re-operation, or conversion to valve replacement due to remnant regurgitation.

**Table 3:** Perioperative annular dimensions in the parasternal long-axis view

	Preop dimensions	Postop dimensions	*P* value
In diastole (max), cm	32.2 ± 7.3	21.1 ± 5.1	<0.0001
In systole (min), cm	27.1 ± 6.7	17.6 ± 4.3	<0.0001
Dynamics, % (max-min/min)	18.8 ± 11.7	19.5 ± 9.3	0.154

*Max* maximum dimension, *min* minimum dimension, *Preop* preoperative. *Postop* postoperative

## Discussion

The PMA strip was designed to lift the middle portion of the posterior annulus and increase coaptation length with placement in the posterior annulus, sparing the anterior annulus and commissures. Two mitral leaflets can yield competent coaptation with reduction of the septo-lateral annular dimensions without reduction of the transverse annular dimensions, particularly in patients with functional MR [7]. Although transverse reduction of the mitral annular dimensions is developed in the usual mitral annuloplasty, a transverse annular reduction is not likely to be a prerequisite for two leaflet coaptation.

The middle part of the strip is lifted by two thick margins into a curvilinear shape. As a result, the middle of the annulus-strip complex is lifted on the base of both commissural planes and the posterior annulus becomes curvilinear with a resultant reduction of the septolateral dimensions. The reduction effect of the septolateral dimensions must be more effective than the usual rings or bands that make a round annular shape. Such a coaptation-enhancing mechanism is seen in the GeoForm annuloplasty ring (Edwards Lifesciences, Irvine, CA, USA) with a diminished antero-posterior distance [8] used to repair ischemic MR, but the motion of the anterior annulus and both commissures is restricted by the rigid ring fixing the annular circumference.

In both commissures spared from the strip annuloplasty, hinges are made by the lower-placed strip ends, which tend to be straightened. Regurgitation or prolapse from the spared commissures did not occur during the follow-up period.

In our cases of rheumatic or ischemic MR or narrow posterior leaflet < 1.0 cm in height, the posterior leaflet was augmented with an elliptical pericardial patch to obtain a sufficient coaptation area prior to PMA [9]. A tethered leaflet causing ischemic MR [10] can yield a proper coaptation area with posterior leaflet augmentation. The leaflet augmentation was also useful in rheumatic MR patients with a short leaflet height. The combined procedure of posterior leaflet augmentation and PMA is a two-leaflet repair rather than a monocusp repair.

MR caused by annular dilatation was well repaired with placement of the strip only. In patients with posterior leaflet prolapse due to chordal rupture, however, new chord placement [6] or patch vavuloplasty [4] may be more effective for increasing the leaflet coaptation area than the resection techniques for the prolapsed segment.

After the typical mitral annuloplasty using a ring, the redundant posterior leaflet with leaflet augmentation may be a risk for developing new systolic anterior motion [11], because the posterior leaflet has been fixed to the trigones by the usual rings or bands. However, because the PMA strip is separated from the anterior annular components, the augmented posterior leaflet has nothing to do with systolic anterior motion.

The size of the annuloplasty rings or bands is determined by various methods [12–14], but the ring size or band length recently has not seemed as important [12, 14, 15]. For our patients, the posterior annulus was reduced to approximately 1.5 times the anterior annular length [16]. The strip length is not likely to affect the anterior annular length after strip annuloplasty.

The PMA is a simple procedure that can be easily performed with six interrupted mattress sutures in the posterior annulus, and its reliable coaptation induction results in a high repair rate without re-repair or conversion to valve replacement. Because of its simplicity and reliability, the procedure can be performed liberally for most MR cases that are a questionable decision for annuloplasty, such as moderate MR associated with dilated annulus, during the other main cardiac procedure. We never experienced hemolysis from remnant regurgitation, probably because of the flat shape of the strips and supraannular position.

The present study has some limitations. We did not obtain three-dimensional echocardiographic images that could demonstrate the lifted posterior annulus and preserved commissural hinge. Also, no comparison study with other rings or bands was performed. In the follow-up computed tomography, however, the lifted posterior annulus and the hinged commissures were readily demonstrated.

## Conclusions

PMA using a novel strip for placement in the posterior annulus showed a sufficient coaptation height secondary to reduction of the septo-lateral annular dimension and dynamic change of the dimensions. It can be considered as an alternative mitral annuloplasty technique with satisfactory results.
